# Transformative potential of conservation actions

**DOI:** 10.1007/s10531-023-02600-3

**Published:** 2023-04-15

**Authors:** Anni Arponen, Anna Salomaa

**Affiliations:** 1 Ecosystems and Environment Research Programme, Faculty of Biological and Environmental Sciences, and Helsinki Institute of Sustainability Science (HELSUS), University of Helsinki, Helsinki, Finland; 2 Research Group Politics of Nature and the Environment (PONTE), Faculty of Management and Business, Tampere University, Tampere, Finland; 3 Unit of Social Research, Faculty of Social Sciences, Tampere University, 33014 Tampere, Finland

**Keywords:** Biodiversity, Conservation interventions, Leverage points, Policy instruments, Sustainability, Transformative change

## Abstract

Transformative change can help achieve the 2050 vision of biodiversity, but concrete ways to achieve it are only being discovered. To contribute to the understanding of the practical options for concrete action to foster, accelerate and maintain the transformative change*,* we assessed the leverage potential of existing conservation actions using the Meadows’ Leverage points framework. We took the actions from the Conservation Actions Classification by the Conservation Measures Partnership. The outcome is a scheme that evaluates at which leverage points, from simple parameters to paradigms, the different conservation actions have potential to make an impact, and thus impact systemic change. We found that all conservation actions have potential to leverage systemic transformative change, with varying coverage of the leverage points. All leverage points were addressed by several actions. The scheme could be used both as an interim tool for evaluating transformative potential in different broad datasets, but also help with planning of new conservation policies, interventions and projects. We hope our work could be a first step toward standardization and broader adoption of assessing leverage in conservation research and practice, achieving broader socio-ecological system leverage with conservation tools.

**Supplementary Information:**

The online version contains supplementary material available at 10.1007/s10531-023-02600-3.

Introduction Sustainability goals for 2030 and beyond may only be achieved through transformative change, as summarized in the Global Assessment Report on Biodiversity and Ecosystem Services by the IPBES. IPBES defines transformative change as “a fundamental, system-wide reorganization across technological, economic and social factors, including paradigms, goals and values” (Intergovernmental Science-Policy Platform on Biodiversity and Ecosystem Services 2019). The Transformative Change Assessment of IPBES aims to understand and identify factors in human society that may be leveraged to bring about transformative change for the conservation, restoration and wise use of biodiversity, while taking into account broader sustainable development goals. Importantly, the assessment has a strong focus on practical solutions to advance transformative change, which have thus far been underrepresented in scientific literature.

Governing transformative change requires understanding of several fields, for example conservation and climate change, and their interactions (Pascual et al. 2022). Recently studies on transformative change in the context of biodiversity actions and IPBES have for example taken important steps to understand the variety of current knowledge producers and what kind of implications this has for content and implementability of the proposed actions (Díaz-Reviriego et al. 2019; Beck and Forsyth 2020; Massarella et al. 2021). Others have proposed general principles and pathways toward transformative conservation (Raatikainen et al. 2021; Fougères et al. 2022), while comprehensive assessments of the transformative potential of biodiversity actions have been lacking.

Various methods and theories examine sustainability transformation and transition (Feola 2015; Salomaa and Juhola 2020). Among them, Meadows’ (1999) leverage points framework (Box 1) draws from systems analysis, describing leverage points as places in complex systems where a small shift may lead to fundamental changes in the system. It is well suited and a pragmatic approach for comprehensively analyzing complex socio-ecological systems, from ecological patterns and processes to goals and paradigms of the society. Even though conceived over two decades ago, it has started to gain popularity in empirical research only lately (Riechers et al. 2022).

**Box 1:** Leverage points are places to intervene in a system, defined by Meadows (1999). Abbreviations in parentheses are used in Table 1.

12. Constants, parameters, numbers (such as subsidies, taxes, standards). (Parameters)
11. The sizes of buffers and other stabilizing stocks, relative to their flows. (Buffers)
10. The structure of material stocks and flows (such as transport networks, population age structures). (Stock structure)
9. The lengths of delays, relative to the rate of system change. (Delays)
8. The strength of negative feedback loops, relative to the impacts they are trying to correct against. (Control loops)
7. The gain around driving positive feedback loops. (Driving loops)
6. The structure of information flows (who does and does not have access to information). (Information)
5. The rules of the system (such as incentives, punishments, constraints). (Rules)
4. The power to add, change, evolve, or self-organize system structure. (System structure)
3. The goals of the system. (Goals)
2. The mindset or paradigm out of which the system—its goals, structure, rules, delays, parameters—arises. (Paradigms)
1. The power to transcend paradigms. (Transcendence)

As conservation is inherently inter- and increasingly transdisciplinary, a leverage points framework for assessing conservation interventions could be enormously beneficial as a methodological boundary object, facilitating collaboration of academics from different disciplines and other societal stakeholders (Fischer and Riechers 2019; Davila et al. 2021). Leverage points can work as a heuristic tool for identifying transformative potential of conservation actions (Davila et al. 2021). Previously leverage points have been used in biodiversity-related research in variable ways, for example, to identify sustainability challenges and opportunities in rural landscapes (Fischer et al. 2022), to support the reframing of restoration ecology as intervention ecology (Hobbs et al. 2011), or to advance evidence-based practice in environmental management (Keene and Pullin 2011). Even though conservation frames and thereby specific goals can vary and be even conflicting with each other (Mace 2014), here we consider the overall aim of conservation to be in line with the SDGs and consider their leverage of the social-ecological system towards sustainability.

Conservation takes place through multiple kinds of actions that vary in their purpose and scope. The Conservation Measures Partnership is a global community of NGOs, government agencies, funders as well as private businesses that strives to improve the effectiveness of conservation through knowledge sharing and standardization. A key product is the Conservation Actions Classification (Salafsky et al. 2008, https://conservationstandards.org/library-item/conservation-actions-classification-v1-0/), which has become a broadly used standard for classifying conservation actions. It comprehensively covers actions from local-scale site and species stewardship measures to financial incentives and ways of promoting behavior change. It is designed to be simple, hierarchical, comprehensive, consistent, expandable, exclusive, and scalable, in other words, well suited for analytical purposes.

Even though transformative change calls for novel, innovative, transdisciplinary solutions, it is also important to understand the place of existing, established conservation approaches in the big picture. Our aims with this piece are twofold. (1) We provide a tentative assessment of the transformative potential of different kinds of conservation actions through the leverage points framework. (2) The resulting scheme can be further applied as an analytical tool for evaluating transformative potential, for example in different broad datasets, but also help with planning of future conservation policies, interventions and projects.

## Material and methods

To represent different kinds of conservation actions as broadly as possible, we used the Conservation Actions Classification v 2.0 by the Conservation Measures Partnership (Salafsky et al. 2008https://conservationstandards.org/library-item/conservation-actions-classification-v1-0/). The full descriptions of the categories are presented in Online Resource 1.

We follow the original framework of Meadows on Leverage Points (LPs) for system change (Meadows 1999) as an analytical frame for assessing the leverage potential of conservation actions. Koskimäki (2021) described these LPs as key *system properties* where focused interventions can induce changes in the system to distinguish from divergent use of the term, e.g. by Chan et al. (2020), whose LPs are largely system outcomes rather than properties. In addition to LPs, we use four LP characteristics groups: parameters, feedbacks, design and intent, by Abson et al. (2017). We define the system under observation as the global social-ecological system, where biodiversity itself and the human actions influencing it are both parts of the same, nested system constituting of different localities and scales. Naturally, the core of our system under observation is biodiversity conservation.

We ask, at which leverage points each conservation action does or potentially could operate? Each action can have multiple kinds of effects and characteristics and hence be associated with multiple LPs. We started by going through the actions and based on our own expertise on conservation policy instruments, conservation effectiveness and system transformation, identified the leverage points that appeared most relevant for each, writing down the reasoning behind our choices. Our previous work with LPs in conservation context (Salomaa and Arponen 2021, forthcoming) and previous studies that have operationalized Meadows’ framework for qualitative coding (Lidgren et al. 2006; Carey and Crammond 2015; Manlosa et al. 2019; Rosengren et al. 2020; Dorninger et al. 2020) guided us in the process. We cross-tabulated the actions and leverage points marking strong potential with a black circle, whereas cases where the association is weaker or depends on the exact method under the action category or is particularly sensitive to quality of implementation or context, we marked with a white circle. The first author made the first draft version, which was iteratively refined in turns, with intermittent discussions. We then used literature to refine our interpretation. We searched for papers related generally to leverage points and conservation first in ISI Web of Knowledge and complemented the literature with additional searches on the different actions. As our purpose was not to perform a systematic review, we did not attempt to cover all possible individual actions and aspects related to them, which due to lack of studies from the leverage points perspective would have required extensive cross-disciplinary reviews on each topic.

Because our scheme is intended to be used as an interim tool for analysis, we chose a pragmatic approach regarding indirect impacts of actions. Leverage points have their origins in the science of complex systems. Similarly, ecology is a science of complex networks, interdependencies, and processes, where narrowly targeted actions may have escalating impacts and feedbacks, easily extending across LP7-12. There may be chains of leverage (Fischer and Riechers 2019) where the impacts on one LP precipitate changes across others: All conservation ultimately aims at halting biodiversity decline, affecting parameters (LP10-12). However, indirect impacts and interdependencies are often difficult to predict and can vary greatly even within different implementations of a single action, and therefore we did not hypothesize for such impacts but limited our classification to more direct and likely leverage points. Instead, we did include impacts that could be considered indirect, but which represented the purpose of the action and could not be associated with other conservation actions, such as change of consumer behavior achieved by Outreach & Communications (3.1.).

Meadows considered maintenance of species’ habitats to represent Negative feedback loops (LP8), because even though encroaching on the habitats of endangered species may not appear to have dramatic immediate consequences, in the long term survival of the whole system is compromised. This describes well the rivet metaphor of biodiversity loss, and following this logic, all conservation actions could ultimately fall under LP8. But in this study, we operate within the realm of conservation, trying to distinguish between different types of actions and their leverage, so it would not be meaningful to adopt this definition here. Meadows also described capacity to evolve as LP4, and here too, all conservation could be seen to contribute toward safeguarding the evolutionary process, but classifying all actions under LP4 would be of little use for comparing them. Thus, we marked only the power to add, change, evolve or self-organize social (social-ecological) system structure under LP4. In a similar vein, all conservation could be considered to delay extinction, but we classified it as LP9 only for the species and ecosystem specific actions where the impact on delaying extinction would be direct and the main point of the action. Regarding LP7, we included both positive feedback loops that favor sustainability as well as positive feedback loops that accelerate unsustainability. The action 3. Awareness Raising is classified under B. Behaviour change, thus we didn’t problematize this knowledge-to-behavior change link in the analysis regarding transformative potential.

## Results

We found that all conservation actions have potential to leverage systemic transformative change. All leverage points were addressed by several actions. At the highest level of organization, the Action classification is in line with Meadows’ leverage points (Table 1): A. Target Restoration/Stress Reduction Actions lead to changes in conservation targets without first reducing threats or creating enabling conditions and are thus expected to operate at shallow leverage points, while B. Behavioral Change/Threat Reduction Actions and C. Enabling Condition Actions are aimed at deeper leverage points. There is, however, much variation among actions within each class, described in more detail below.

**Table 1:** Strong/weak transformative potential of conservation actions indicated by black/white circles. The potential can be weak either by default, because it depends on quality of implementation, or because it applies only to some specific actions within the action category. Full names of the leverage points are given in Box 1. Sustainability leverage points by Meadows (1999) and system characteristics by Abson (2017)

System characteristics	Parameters			Feedbacks			Design			Intent		
Leverage points	LP12 Parameters	LP11 Buffers	LP10 Stock structure	LP9 Delays	LP8 Control loops	LP7 Driving loops	LP6 Information	LP5 Rules	LP4 System structure	LP3 Goals	LP2Paradigms	LP1 Transcendence
*A. Target restoration/stress reduction actions*												
1. Land/water management												
1.1 Site/area stewardship	⚫	⚪	⚪									
1.2 Ecosystem & natural process (Re)creation	⚫	⚪	⚫	⚫	⚫	⚪						
2. Species management												
2.1 Species Stewardship	⚪	⚪	⚪	⚪								
2.2 Species re-introduction & translocation	⚪	⚪	⚪	⚪		⚪						
2.3 Ex-situ conservation	⚪	⚪	⚪	⚪								
*B. Behavorial change/threat reduction actions*												
3. Awareness raising												
3.1 Outreach & communications	⚪	⚪	⚪	⚪	⚪	⚪	⚫	⚪	⚪	⚪	⚪	⚪
3.2 Protests & civil disobedience	⚪	⚪	⚪	⚪	⚪	⚪	⚫	⚪	⚪	⚪	⚪	
4. Law enforcement & prosecution												
4.1 Detection & arrest	⚪	⚪	⚪		⚪	⚪	⚪					
4.2 Criminal prosecution & conviction					⚪	⚪	⚪	⚪				
4.3 Non-criminal legal action					⚪		⚪	⚪	⚪	⚪		
5. Livelihood, economic & moral incentives												
5.1 Linked enterprises & alternative livelihoods	⚫	⚪	⚫		⚫	⚪			⚪			
5.2 Better products & management practices	⚪	⚪	⚪									
5.3 Market-based incentives	⚫	⚪	⚫		⚫	⚪	⚪	⚪				
5.4 Direct economic incentives	⚫	⚪	⚫		⚪	⚪		⚪				
5.5 Non-monetary values	⚪	⚪	⚪							⚫	⚫	
*C. Enabling condition actions*												
6. Conservation designation & planning												
6.1 Protected area designation &/or acquisition	⚫	⚫	⚫	⚪	⚪	⚪		⚪				
6.2 Easements & resource rights	⚪	⚪	⚪	⚪	⚪	⚪		⚪				
6.3 Land/water use zoning & designation	⚪	⚪	⚪	⚪	⚪	⚪		⚪				
6.4 Conservation planning							⚫					
6.5 Site infrastructure	⚫	⚪	⚪									
7. Legal & policy frameworks												
7.1 Laws, regulations & codes	⚫	⚫	⚫		⚪		⚫	⚫	⚪	⚪	⚪	
7.2 Policies & guidelines	⚪	⚪	⚪		⚪		⚪	⚪	⚪			
8. Research & monitoring												
8.1 Basic research & status monitoring					⚪		⚫					
8.2 Evaluation, effectiveness measures & learning					⚫		⚫					
9. Education & training												
9.1 Formal education							⚫		⚪		⚪	⚪
9.2 Training & individual capacity development							⚫		⚪		⚪	⚪
10. Institutional development												
10.1 Internal organizational management & administration	⚫	⚫	⚫		⚪	⚪						
10.2 External organizational development & support					⚪		⚪		⚪			
10.3 Alliance & partnership development							⚪		⚪	⚪	⚪	
10.4 Financing conservation	⚫	⚫	⚫		⚪	⚪						

### Land/water management

Site/Area stewardship (1.1) are small-scale actions with a physical character: enhancing viability or mitigating stress implies changing a quantity (LP12). Whether they affect buffers (LP11) depends on how ambitious goals are set for the actions. Ideally, conservation actions should always aim at adequate buffers rather than minimum indispensable quantities, while in practice the ongoing population declines show that this often fails. Some actions may affect local spatial structure of habitat, such as fencing, or population structure through genetic manipulation (LP10).

Ecosystem and Natural Process (Re)Creation (1.2) instead affects broader entities and feedbacks by definition. These actions eventually influence the amount of habitat, or size of population (LP12), even though through affecting a process. Process (re)creation also should consider stocks and flows, how they are stabilized (LP11) and structured (LP10). E.g., a functioning hydrological system is all about flows and buffering. Structure is critical for (re)creating processes: From structural components such as retention trees, to overall age structure of a forest, or spatial connectivity of habitat. Actively restoring instead of letting nature take its course reduces delays in recovery, e.g. when infilling ditches in drained peatlands instead of just stopping their maintenance (LP9). Restoration can target and bring back natural regulatory ecosystem functions (LP8, Hobbs et al. 2011) for example in the food web, for flood control, or for regulating microclimatic conditions. Restoration activities can themselves have a regulatory role when addressing ongoing stresses, for example, mowing of wet grasslands changes vegetation composition and growth rate, attracting more water birds, thus keeping the amount of their habitat at an acceptable (safe) level (Angelstam et al. 2022, LP8). They may also provide gains around driving positive feedback loops (LP7) if the action effectively addresses e.g. eutrophication, spread of invasives, habitat fragmentation or other processes that have a self-reinforcing negative impact on biodiversity (Hobbs et al. 2011; Davila et al. 2021), or by enabling a self-reinforcing process that increases biodiversity.

### Species management

Species Stewardship (2.1) actions aim to keep species or populations viable without considering broader ecosystem impacts, targeting numbers and parameters (LP12). This is often done by influencing further numbers and parameters (LP12), such as increasing availability of resources in different ways, reducing the amounts of nutrients, reducing the prevalence of a disease by vaccinating individuals, and so forth. Whether these actions amount to buffering or stabilizing effects, depends on their level of ambition: Maintaining more than the minimum breeding population of an endangered species was one of Meadows’ original examples of LP11. Local scale actions can impact Structure of material stock and flows (LP10) especially for smaller organisms, but also more generally when the actions are well coordinated and planned, for example population age structure (an original example by Meadows, although referring to human populations), metapopulation dynamics and movement/migration patterns. Extinction tends to happen with a delay with respect to its causes, and species stewardship actions can further prolong this delay (LP9) giving time for more fundamental, deeper leverage changes to take place. They can also boost population growth and colonization to new sites that otherwise might happen slowly (or not at all), shortening the delay in species recovery after a stressor. For instance, providing nest boxes for birds in a managed forest until the forest matures enough to become suitable for cavity-nesters, would reduce the delay in species (and ecosystem) recovery.

When Species Re-Introduction & Translocation (2.2) is done to save the species itself, leverage remains relatively low, affecting the species’ own (local) population size or distribution (LP12). Again, if it is done with ambitious enough goals, so that the resulting population size is large enough to be resilient against unexpected events, it will affect LP11. As translocations must be planned per specific locations and individuals, they are more likely to influence structure positively: both spatial as well as population structure. For example, translocations can be planned to improve genetic diversity of a population, or to a location that connects isolated populations (LP10), in which case a focused effort can lead to a gain around a driving positive feedback loop (LP7) through genetic rescue, provided that population genetics of both the target population and translocated individuals are known. Such strategic translocations can be challenging endeavors for their data requirements and difficult practical implementation, but when successful, they have a strong gain around this driving positive feedback loop, however, limited to the scale of the targeted species itself. Translocations have the potential to reduce delays with respect to natural dispersal (LP9), even though in reality due to high costs and information requirements, resources may be concentrated on species that have poor or nonexistent chances of natural dispersal to the targeted location.

Ex-situ Conservation (2.3) clearly influences species specific numbers, increasing the number of extant individuals and their reproductive rates (LP12). Maintaining viable populations of species in captivity, as well as gene banking, represent a type of buffering approach by definition (LP11). Maintenance of genetic diversity directly affects the genetic structure of stocks, and the carefully regulated breeding in zoos also affects population age/sex structure (LP10). Ex-situ conservation can also prolong the delay before extinction (LP9).

### Awareness raising

The primary channel of leverage for Outreach & Communications (3.1) is self-evidently through the structure of information flows (LP6). Outreach methods vary greatly in their transformative potential, from shallower leverage of simple newspaper articles to the deeper leverage of immersive and experiential approaches. Outreach may or may not be effective, but when it successfully leads to behavior change, it affects several leverage points. For example, shifting diets toward plant-based protein would affect parameters (LP10-12), but also enable reforming the structure and rules of the entire food system (LP4-5). Strengthening material links with nature locally would shorten Feedbacks and Delays (LP8, LP9) by reducing the externalization of environmental impacts of consumer choices (Abson et al. 2017; Carrasco et al. 2017). Reporting on conservation success stories that have brought benefits to the local community can encourage more active participation, forming a reinforcing feedback loop (LP7) around the conservation action itself (Raatikainen et al. 2020; Angelstam et al. 2022). Successful outreach campaigns can also empower and increase people’s sense of agency (Linnér and Wibeck 2021; Wamsler et al. 2022) affecting the power to change system structure (LP4). Outreach that reinforces nature connectedness also has the potential to influence the system paradigm and goals (LP2, LP3, Abson et al. 2017; Ives et al. 2018; Raatikainen et al. 2020; Richardson et al. 2020), leading to more sustainable behaviors and increased nature contact, reinforcing its own impact (LP7, Barragan-Jason et al. 2022). Well-designed outreach through transdisciplinary, participatory approaches, could even enlighten the target audience regarding co-existence and value of worldviews beyond their own, inducing transcending paradigms (LP1, Raatikainen et al. 2020; Linnér and Wibeck 2021). Even though lobbying decision makers is considered under Legal & policy frameworks (7), they too are susceptible to general public outreach efforts, which can influence the broader political agenda. Increased awareness can influence rules of the system (LP5), for example, the Red List assessments have influenced legislation in Finland (Salomaa and Arponen, forthcoming), or even catalyze system structure change (LP4). Similarly, increased awareness among decision makers may also influence system goals (LP3), such as the SDGs.

Protests and civil disobedience (3.2) can influence Parameters (LP10-12). Classical examples are camp-outs of activists that have stopped logging or other destruction of the environment, resulting even in establishment of protected areas. (Note that boycott is considered an economic incentive in the Action classification). Protests and civil disobedience have the potential to alter rules (LP5) and speed up decision making and implementation of conservation actions that are often delayed by tortuous legal and administrative paths (LP9). There can be gains around both types of feedback loops if protesting successfully stops or prevents a self-reinforcing threat or driver of biodiversity loss (LP7) or maintains them at a sustainable level (LP8). Protest and civil disobedience can be effective in bringing conservation issues into public awareness (LP6) by drawing media attention. In addition, investigative journalism and naming and shaming campaigns can uncover hidden information and increase accountability (LP6). Civil disobedience has also been recognized to increase people’s capacity to self-organize (LP4, Priebe et al. 2022). Policy dismantling and even purposeful destabilization of institutions has been cited as a powerful, albeit risky lever (Abson et al. 2017), possibly extending the impact of activism to goals and paradigms (LP2, LP3).

### Law enforcement and prosecution

Detection & Arrest (4.1) have immediate effects on parameters (LP10-12), when for example controlling poaching directly influences population sizes, or surveillance for violations of environmental laws affect parameters regarding pollution rates, erosion, etc. Poaching and trafficking can be biased toward mature (male) individuals affecting population structure. Some criminal activities are difficult to control, but when successful, the deterrent effect would act as a negative, controlling loop (LP8). Sad examples of this were seen when during the Covid pandemic patrolling in protected areas ceased and illegal activities such as burning and poaching increased, however, this was a synergistic effect with increased poverty (Anagnostou et al. 2021; Eklund et al. 2022). Indeed, the effectiveness of deterrence will largely depend on case-specific social and economic factors, for example, livelihoods depending on exploitation will weaken it (Moreto and Gau 2017). Demand for illegal wildlife products is flexible, prices going up with reduced supply, forming a perverse incentive (LP7) that reduces the effectiveness of law enforcement, nonetheless, Detection & Arrest can be a part of a solution that eventually breaks the cycle. Detection & Arrest are also a part of the process of exposing illegal activities (LP6).

Criminal Prosecution & Conviction (4.2) also have one kind of deterrent function (LP8, LP7). Collecting evidence and investigating illegal activities form new flows of information, as does exposing them to the public (LP6). Although the power of courts varies from country to country, precedents can sometimes determine new rules, or courts can ask for preliminary rulings from a superior (national or international) court, which become binding (LP5, Hill and Martinez-Diaz 2019).

Non-Criminal Legal Action (4.3) contains judicial reviews and such that act as regulating loops, keeping the impacts of harmful activities within acceptable boundaries (LP8). Non-criminal legal actions can be important in generating new channels of information flow and for increasing accountability for biodiversity loss (LP6). As above, Non-Criminal Legal Action can affect Rules through precedents or preliminary rulings (LP5). Class actions are classically considered to be strongly empowering for the class members (Erichson 2003), and indeed, they have a character similar to public movements, civil disobedience and protest, where people assume collective power which may lead even to changing system structure (LP4). Climate change litigation is well ahead of biodiversity focused cases, but evidence based on the rapidly growing number of climate related cases suggests that successful litigation against a government, especially when strategically targeting inadequacy of policies and action (‘systemic litigation’), could influence system rules (LP5, Hill and Martinez-Diaz 2019) and goals (LP3, Setzer and Higham 2022). Their long-term impacts remain to be discovered in the future.

### Livelihood, economic and moral incentives

Linked enterprises and alternative livelihoods (5.1) initiatives would have direct Parameter impacts on targeted biodiversity (LP12), and depending on their arrangement, possibly also on Buffers (LP11). They will impact entrepreneurship and labor market structure (LP10). Linked livelihoods that directly depend on the maintenance of natural resources have a strong regulating character regarding biodiversity (LP8) but a self-reinforcing impact through increased revenue, societal interest and support for the livelihood is also possible (LP7, Angelstam et al. 2022). Provision of alternative livelihoods could potentially end destructive activities/industries altogether (LP7), although they are more likely to reduce and regulate the impacts. Providing a more diverse set of alternatives for livelihoods could give room for the system structure to self-organize (LP4).

Adopting Better products and management practices (5.2) influences parameters (LP10-12). Seal-friendly fishing gear, greening of supply chains of corporations, or swapping for low water-use crops do not aim at any deeper change in the society, just a less damaging way of proceeding with business-as-usual.

Financial incentives, when implemented within existing structures, affect mainly parameters (Abson et al. 2017). Market-Based Incentives (5.3) contain a highly diverse set of actions from the perspective of leverage. They can directly hit consumption parameters (LP12, depending on design also and LP10 and LP11), and affect market structure (LP10), but also feedback mechanisms are commonly involved. Environmental markets (carbon markets, ecological compensation schemes) would provide controlling feedback loops through internalizing costs (LP8, Meadows 1999), while well targeted and substantial enough green financing has the potential to arrest escalating biodiversity loss processes or threats (LP7, funding for public health in Carey and Crammond 2015). Certification schemes affect information flows from producer to consumer (LP6, Dajka et al. 2020). Environmental markets represent new rules, as do certification schemes at a different level (LP5).

Direct Economic Incentives (5.4) were among the original examples of LP12 in Meadows’ work, for example, compensation for damage caused by large carnivores has been described to affect LP12 (Hartel et al. 2019), but depending on how they are designed, they can also affect buffers (LP11) and they inevitably affect structure of stocks and flows (LP10)—such as market structure distortions in favor of biodiversity friendly products. Direct economic incentive schemes can provide regulating feedback loops, provided they are well targeted and responsive to current needs (LP8). An interesting reinforcing loop structure was reported by Angelstam et al. (2022), where incentives increased land owners’ willingness to take part in wetland management, which helped develop nature tourism and bring prosperity to the area, which in turn triggered more incentives from the municipality (LP7). Some schemes, such as the EU’s Agri-environment schemes or payments for voluntary conservation, have reshaped The rules of the system (LP5), although such rules are weaker than laws and punishments (Meadows 1999).

There are some non-monetary values (5.5) that can be quantified, such as health benefits (Aerts et al. 2018), thus having parameter impacts (LP10-12), but because the purpose of this action by definition is to use intangible and moral values to change behaviors and attitudes, we consider their importance for transformative change to arise primarily from providing alternative system goals and paradigms (LP2-3).

### Conservation designation and planning

Protected area designation and/or acquisition (6.1) focuses mainly on parameters (Abson et al. 2017). Constants, parameters and numbers (LP12) are affected by increasing the amount of area protected (Meadows 1999). Area targets should be adequate to buffer against fluctuations (LP11) and spatial structure of the protected area network should be taken into account (LP10) or otherwise biodiversity will eventually decrease within them (LP9). Formal protection of sites can address self-reinforcing threats, such as habitat fragmentation (LP7), as well as mitigate and regulate ongoing stressors (LP8). This category covers the establishment of protected areas legally (LP5).

Easements & Resource Rights (6.2) as well as Land use zoning and designation (6.3) have in principle all the same aspects as PAs but with lesser ecological impact because they are addressing just some aspect of the location, providing a lower degree of protection than IUCN protected area categories I–IV (LP7-12, LP5).

Conservation planning (6.4) deals with designing and planning actions, but not with their implementation, therefore its direct impact manifests at The structure of information flows (LP6) independently of how/whether the information will flow into practice. When used in combination with another action, it increases their quality of implementation and thus leverage.

Site Infrastructure (6.5) investments mainly belong to Constants, parameters and numbers (LP12), while LP10-11 can be involved depending on details of implementation.

### Legal and policy frameworks

Laws, Regulations & Codes (7.1) as well as Policies & Guidelines (7.2) can have highly variable paths of influence, depending on what issues they address. Laws, regulations, and codes are more binding than policies and guidelines. A direct impact on parameters (LP10-12) is possible, for example through legal protection of species that protects them from exploitation, or through timber harvest quotas (Meadows 1999), and here the bindingness makes parameter-level transformative potential of laws stronger than that of policies. Deterrence is usually considered in the context of getting caught and convicted (4.1–4.2), but for the regular law-abiding citizens knowing that something is illegal or strongly advised against may be enough to prevent doing it (LP8). These categories (7.1, 7.2) include educating or lobbying lawmakers and policymakers, affecting the Structure of Information flows (LP6). Self-evidently these categories affect Rules (LP5). Transformative potential of laws on LP6 and LP5 is stronger than that of policies. New legislation (7.1) and new policies (7.2) can also enable system structure change (LP4), for example, a requirement for ecological compensation could generate a whole new ensemble of actors and markets with their feedback mechanisms, which previously did not exist. The concept of ecocide in international criminal law, framed as comparable to genocide or crimes against humanity, is also gathering momentum. Being independent of whether harm will come to humans, it is a strongly ecocentric concept, and holds promise for deeper societal impact, including a paradigm shift (LP2, White and Kramer 2015). It is based on the concept of earth stewardship, which implies shifting also system goals (LP3, Chapin et al. 2022).

### Research and monitoring

Basic Research and Status Monitoring (8.1) inevitably affect the structure of information flows (LP6). Meadows lists monitoring systems as an example of a controlling feedback loop (LP8). Scientific knowledge can contribute to behavior change, but those impacts are addressed in more detail under Public outreach. In addition to LP6, Evaluation, Effectiveness Measures and Learning (8.2) about the effectiveness of conservation work e.g. via adaptive management or double-loop learning forms a strong regulating feedback loop to practice (LP8, Meadows: monitoring systems, Keene and Pullin 2011).

### Education and training

Formal Education (9.1), in the Action classification referring to specialized education on conservation, addresses information flows (LP6). Education can provide empowerment and promote agency, which enables system structure change (LP4, Linnér and Wibeck 2021; Sidiropoulos 2022). Conservation education that teaches about different alternative philosophical perspectives can impact even paradigms (LP2) or enable transcending them (LP1) (Moon and Blackman 2014; Pascual et al. 2022). Similarly, Training & Individual Capacity Development (9.2) addresses information flows (LP6). Both can include capacity building which affect the power to change system structure (LP4) and in some cases even paradigms and transcending them (LP2 and LP1).

### Institutional development

Internal Organizational Management & Administration (10.1) handles both human and material resources for conservation organizations (LP10-12), and all actions for Financing Conservation (10.4) influence parameters regarding money (LP10-12). Regarding 10.1 and 10.4 the Covid-19 pandemic highlighted the importance of financial buffers (LP11) as well as diversification of funding structure for protected areas (LP10). Many were suddenly in deep trouble as tourism revenues seized and there were no buffers or alternative funding sources (Waithaka et al. 2021). Funding structure diversification may also release the organization from a self-reinforcing loop where they feel pressure to enact the goals of the funder (LP7, Berl et al. 2022). These issues are not implicitly addressed by any fundraising action, but rather, they should be in a key role when planning for overall financing (10.1). Financing (10.1. and 10.4) can also be seen as a regulatory loop against biodiversity loss (LP8) and if investments are targeted effectively and proactively, they may also reinforce or create new positive feedback loops (LP7, Carey and Crammond 2015 on funding for public health).

External Organizational Development & Support (10.2) will strengthen organizations ensuring continuity in the regulatory role they have (LP8). External support to an organization can include for example consulting services, forming a new channel of information flow (LP6). Creation of new environmental organizations changes system structure (LP4).

The impact of Alliance and Partnership Development (10.3) depends largely on the context and who is involved and what is done in the partnership, but all kinds of collaborations would be expected to influence the structure of information flows (LP6), and especially so when the collaboration is about knowledge creation (Keene and Pullin 2011). Impact on system structure is also possible (LP4, Keene and Pullin 2011; Hartel et al. 2019; Burgos-Ayala et al. 2020). Collaborative partnerships in pastoral social-ecological systems have been reported to succeed in changing paradigms to reconstruct power relations, build relationships and change mental models (LP2, Reid et al. 2021). Convening meetings of local stakeholders is mentioned as an example in this class, so we assume all forms of stakeholder collaboration, including conservation conflict management, would belong here. When performed well, conflict management is a potentially powerful enabling action that can reveal and address the underlying social, psychological, and systemic drivers of conflict (Madden and McQuinn 2014), thereby reaching even system goals and paradigms (LP2-3).

## Discussion

According to our assessment of the transformative potential, all conservation actions have potential to leverage systemic transformative change. Awareness raising strategies, appealing to non-monetary values to change behavior, as well as education and training are the actions that have the potential to operate at the deepest level of leverage, at the level of intent. The broadest coverage over different leverage points (> 7) is attained by awareness raising, market-based incentives, protected area designation and legal frameworks. Actions with most leverage points with strong potential were Laws, Regulations & Codes (5 LPs) and Ecosystem & Natural Process (Re)Creation (4 LPs).

Further research is needed to understand to what extent a successful strategy for systemic transformation would require triggering diverse leverage points (Abson et al. 2017). Which actions would be most impactful in different contexts is also yet to be seen, but past studies have provided broader recommendations for improving the transformativeness of conservation that our research on actions complements. Fougères et al. (2022) recommend the systems approach to which leverage points framework belongs, and linking societal with inner transformation (LP2 with LP1). These principles should be operationalized for conservation planning and further to action, while maintaining adaptivity of actions and governance. In addition, Fougères et al. recommend partnering with political movements to achieve just transformation. Indeed, conservation should build on principles of transformative governance, being integrative, adaptive and equitable, to achieve its transformative potential (Pascual et al. 2022).

Our scheme is certainly not the only way to interpret leverage of conservation actions (note esp. Chan et al. 2020 in the IPBES context) and we present our work as a tentative classification, hoping it will stimulate further discussion and development. Below we discuss some issues that should be kept in mind while applying it to practice.

Our approach should be used only when knowledge on realized leverage of actions toward sustainability is unavailable. It is useful especially for analyzing large datasets, where it would be difficult to scrutinize each action individually regarding their leverage. The resolution is instead inadequate for detailed study of individual conservation policies and projects for several reasons. Sometimes shallow leverage actions can interact with and trigger changes in deeper leverage points (Abson et al. 2017; Fischer and Riechers 2019), and such effects would remain unobserved without more detailed analyses. For example, although direct economic incentives mainly focus on parameters (LP10-12), it is also possible that they may affect the mindset of the involved actors, having an upstream impact on intent leverage points (Abson et al. 2017).

As indicated above, there is also strong contextuality to transformative potential of conservation actions, but even though the leverage points framework is transferable across contexts (Fischer & Riechers 2019), our general level assessment does not in itself account for differences for example in the state of nature, quality and organization of governance, or socio-economic, cultural or historical contexts that may influence what can be achieved with each action in a specific location and scale. Engaging stakeholders to implement restoration actions could reach deeper leverage points than the same actions implemented by public authorities, but even more so if they were engaged in a co-creation process that results in new knowledge, decisions and consequently more impactful actions (Davila et al. 2021; Pascual et al. 2022). The notion of cross-sectorality of transformative change applies beyond the context of knowledge production (Pascual et al. 2022)—for example, cross-sectoral Alliance and partnership development (10.3) could be a much more powerful action than collaboration among different conservation NGOs (Hartel et al. 2019). These are all issues that should be considered when assessing the transformative potential of conservation actions.

The quality of applying the action to practice also matters. High quality data and methods combined with meticulous implementation can make a large difference in the leverage of an action. Actions may also have characteristics that do not directly match with this general scheme. There is also variation in leverage within action categories, for example, the category Outreach & communications (3.1) contains methods from simple newspaper articles to ways of enhancing nature connectedness, which are unlikely to have similar impacts. Continuing the work of collecting evidence at the level of individual actions under the categories would be useful.

The choice of excluding indirect effects from our scheme must be taken into account. It is critical, especially when attempting quantitative assessments of impacts at each leverage point, to consider the indirect impacts explicitly in the analysis, or one may risk underestimating the role of the shallow leverage points. For example, lobbying that leads to changing a law, operates at Information flows (LP6) and Rules of the system (LP5), but the downstream impacts depend entirely on what the law is about. Setting hunting restrictions for a species has very different leverage from a law that obligates companies to compensate their environmental impacts. Another issue is the scale of impact, that is not inherently included in the leverage points framework. How to compare e.g. translocation of a single species that intervenes with a driving positive feedback loop (LP7) vs. adopting globally a new technological solution that affects parameters only? In practice, the user should pay special attention to the points with weak transformative potential (white circles in Table 1), where the association may be specific to only certain actions in the class, or depend on the context or target.

As in the Action classification, we too consider protected areas to deserve a few additional words due to their central role in conservation. Even though their establishment does not stand out in our table as a particularly powerful tool to leverage transformative change, it should be recognized that it is a perfect example of how leverage will depend on multiple synergistic actions and context. An isolated, small protected area located based on convenience, and abandoned on its own to become a ‘paper park’ is very different from an international network of high-quality areas identified based on spatial prioritization, with associated management plans and surveillance, public outreach, education and research taking place within it, and secured and diversified funding for implementing all of it in cross-sectoral international collaboration. Nearly all the actions could be employed to make the most of area protection, covering each and every leverage point. Even though mainstreaming of conservation to all sectors of the society is desperately needed under the current circumstances, protected areas do still maintain their fundamental role in conservation, also from the viewpointt of leverage for systemic transformative change. All of this also goes to show that our pragmatic choices for the analysis do not imply that indirect effects would be of lesser importance for transformative change, but rather that care should be taken to account for all of them separately.

Similar to our work, the Conservation action classification is not without its own assumptions and pragmatic choices. For example, it is stated in the description that establishment of protected areas was considered so central to conservation that it got its own entry, even though technically it could have been a subset of 7. Legal & Policy Frameworkss. Some categories have rather fuzzy borders, for example, whether grazing of meadows belongs to site stewardship or ecosystem and natural process recreation is a matter of deciding the spatial scale of what is considered a landscape. These choices will have repercussions that should be accounted for when applying our scheme on real actions.

## Conclusion

We found varied transformative potential in all of the considered conservation actions. Our results reflected the diversity of the available actions, ranging from the shallow leverage of local site management to the deep impacts of outreach and intangible incentives. The range of leverage points affected may turn out to be a key factor in successful transformations, hence our scheme could be valuable for facilitating extensive analyses of past conservation efforts as well as for planning new ones.

The leverage points framework shows promise for advancing transformative change. There is a rising trend of interest towards its use, which emphasizes the need to operationalize and standardize its use for applied research, also responding to the calls of the IPBES Transformative Change Assessment to develop practical solutions to advance transformative change. There is a need for novel transformative solutions, but it is equally important to increase our understanding of the transformative potential of existing solutions in order to capitalize on their full potential, and distribute our efforts and resources in a way that will best promote transformative change. We hope our work could advance assessing leverage in conservation research and practice, achieving broader socio-ecological system leverage with conservation tools.

## Supplementary Information

Below is the link to the electronic supplementary material.

Supplementary file1 (DOCX 43 KB)

## Data Availability

The study is based on openly available data at https://docs.google.com/spreadsheets/d/1i25GTaEA80HwMvsTiYkdOoXRPWiVPZ5l6KioWx9g2zM/edit#gid=874211847.
