# Supplementary material for: Transformative potential of conservation actions
Source: Biodivers Conserv. 2023 Apr 15:1–23. Online ahead of print. doi: 10.1007/s10531-023-02600-3 (PMC10105141; doi:10.1007/s10531-023-02600-3)
Supplement: Supplementary file 1 — Supplementary file1 (DOCX 43 KB) [file 10531_2023_2600_MOESM1_ESM.docx]

**Transformative potential of conservation actions**

**Biodiversity & Conservation, Anni Arponen & Anna Salomaa**

Anni Arponen, Ecosystems and Environment Research Programme, Faculty of Biological and Environmental Sciences, and Helsinki Institute of Sustainability Science (HELSUS)

P.O. Box 65, 00014 University of Helsinki, Finland

anni.arponen@helsinki.fi

**Online Resource 1**

Conservation Actions Classification v 2.0 by the Conservation Measures Partnership (<https://conservationstandards.org/library-item/conservation-actions-classification-v1-0/>, retriewed 30/11/2022).

| **Classification Levels** | | | ***(Beta version - subject to change)* Proposal for Comprehensive Level 3** | **Definition** | **Exposition** |
| --- | --- | --- | --- | --- | --- |
| **1** | **2** | **3 *(Examples, not comprehensive)*** |  |  |  |
| **A. TARGET RESTORATION / STRESS REDUCTION ACTIONS** | | |  | Actions to directly restore a target or mitigate a stress | These actions lead directly to changes in conservation targets without first reducing threats or creating enabling conditions. |
| **1. Land / Water Management** | | |  | Actions directly managing or restoring sites, ecosystems and the wider environment | This class contains "on-the-ground" conservation actions at specific sites or for broader ecosystems and areas. The term "ecosystem" here is roughly equivalent to the term "habitat" as used by species managers. |
|  | **1.1 Site/Area Stewardship** | | Restoring targets or mitigating stresses via... 1 Mechanical actions (eg removing invasives, mowing) 2 Chemical actions (eg herbicides, liming ponds) 3 Biological control (eg oil eating microbes)  4 Sensory control (eg predator urine, noise deterrents) 5 Separation / border actions (eg fencing, gating caves) 6 Ecological management (eg prescribed fire) 7 Genetic manipulation (eg sterile males of an invasive species) 8 Visitor management (eg routing trails around wetlands) | Enhancing viability / mitigating stresses for sites and/or ecosystem targets, especially on a smaller scale | Includes many different types of "site management" actions. Existing invasive species, pollution, and geological/climate events are treated as stresses here, even though these factors are often categorized as direct threats. This category is meant for actions undertaken by the project team itself (eg conducting controlled burns); actions designed to get other managers to undertake conservation actions (eg training or incenting a rancher to conduct controlled burns) belong in various other categories below. |
|  |  | *cutting invasive vines off trees, liming acid ponds, using microbes to clean up oil spills, use of noise makers to scare off birds, gating caves to protect hibernating bats, routine maintenance in a protected area, use of traditional fire management practices by indigenous peoples, releasing daughterless male mosquitoes, managing park visitors to reduce disturbance* |  |  |  |
|  | **1.2 Ecosystem & Natural Process (Re)Creation** | | Building or restoring key ecosystem... 1 Structural components 2 Abiotic functions & processes 3 Biotic functions & processes | Restoring missing or severely degraded ecosystems and ecosystem functions and processes, especially on a large scale | To some degree, the difference between *1.1 Site / Area Stewardship* and *1.2 Ecosystem & Natural Process (Re)Creation* is a question of scale; 1.1 describes site management whereas 1.2 describes large-scale management efforts. For example, although maintaining a few water control structures could be a site management task, in general we propose that large actions to restore degraded hydrological regimes go here. |
|  |  | *creating forest corridors, grasslands or mangroves on degraded lands, creating artificial oyster reefs, breeching levees to restore wetlands, managing dams to simulate natural hydrological regimes, using livestock to simulate herbivore grazing* |  |  |  |
| **2. Species Management** | | |  | Actions directly managing or restoring specific species or taxonomic groups | This class contains direct conservation actions targeting specific target species or taxonomic groups. Thus, dam breaches aimed at one or more salmon species fit here in *2. Species Management,* whereas dam breaches aimed at restoring general stream connectivity fit in *1. Land / Water Management.* |
|  | **2.1 Species Stewardship** | | Assisting specific taxa *in-situ* via... 1 Population management 2 Shelter management 3 Nutrient / water management 4 Reproduction management 5 Disease / injury management 6 Movement / migration management 7 Interspecific interaction management | Enhancing viability of / mitigating stresses to specific taxa within their current range | Direct work to manage specific species or taxonomic groupings *in-situ.* This category is meant for actions undertaken by the project team itself (eg mowing grassy areas at times to avoid disturbing breeding birds); actions designed to get other managers to undertake conservation actions (eg training or incenting a rancher to use these better mowing practices) belong in various other categories below. Actions focused on managing invasive species that affect a habitat belong in *1.1 Site/Area Stewardship*. |
|  |  | *culling ungulates, heating bat roosts to reduce white-nose syndrome effects, artificial bird nesting boxes/platforms, supplementary feeding, planting food trees, manual pollination of orchids, vaccinating wild dogs, guiding elephants on migrations, shooting or trapping invasive competitors, using false brood parasite eggs to reduce brood parasitism* |  |  |  |
|  | **2.2 Species Re-Introduction & Translocation** | | Facilitation of... 1 Reintroduction of species where they previously existed  2 Translocation of species to new places (benign introduction) 3 Transfer of individuals or genes to increase genetic diversity | Transferring species or genetic material to places where they formerly occurred or to suitable future habitat or benign introductions of species to an ecosystem | Transferring a species in service of conservation of the species itself. Introducing a species in service of ecosystem restoration could arguably go in *1.2 Ecosystem & Natural Process (Re)Creation.* |
|  |  | *re-introduction of wolves, translocation of species imperiled by climate change to new suitable locations, benign introductions of top predator fish to an ecosystem, transferring monkeys to improve genetic diversity* |  |  |  |
|  | **2.3 *Ex-Situ* Conservation** | | Providing *ex-situ* protection to species via... 1 Support within the life of an individual 2 Support / captive breeding over generations 3 Gene banking (eg seeds, sperm, DNA) 4 Genetic reconstruction (eg restoring extinct species) 5 Synthetic species creation | Protecting specific taxa in artificial settings with the aim of ultimately restoring them to their natural settings | To be a true restoration strategy, this approach requires not just doing *ex-situ* conservation, but also ensuring that the taxa are ultimately put back into the wild in the appropriate habitats. To this end, the educational function of zoos & aquaria belongs in *3.1 Outreach & Communications*. |
|  |  | *off-site rehabilitation of injured raptors, captive rearing of mussels for reintroduction, captive breeding of an endangered frog to avoid disease, seed banking, genetic restoration of mammoths from DNA, restoration of tortoises from back-crosses, synthetic species creation* |  |  |  |
| **B. BEHAVORIAL CHANGE / THREAT REDUCTION ACTIONS** | | |  | Actions to reduce direct threats or increase positive behaviors | These actions either change human behaviors that threaten conservation targets or enhance human behaviors that contribute to conservation. |
| **3. Awareness Raising** | | |  | Actions making people aware of key issues and/or feeling desired emotions, leading to behavior change | This class contains actions designed to make people aware of the impact of human activities on conservation targets and/or feel emotions that will lead to the desired behavioral change. |
|  | **3.1 Outreach & Communications** | | Raising awareness via... 1 Reported media (eg newspapers, TV, radio, curated blogs) 2 Social media (eg Facebook, personal blogs) 3 Ads & marketing (eg mail campaign, celebrity media ads) 4 Displays (eg museum or zoo exhibits, park signs) 5 Art (eg paintings, recorded music) 6 Performances (eg puppet shows, theater) 7 Person-to-person engagement (eg info booth, peer mentor) 8 Experiential learning (eg nature walks, outdoor education) | Promoting desired awareness and/or emotions and subsequent behavior change by providing information to target audiences through appropriate channels | Includes "training" or "education" that is primarily designed to get folks to change a behavior (eg educating homeowners to plant native rather than non-native species), but trainings that teach specific skills (eg how to do controlled burns) fit in *9.2 Training & Capacity Development*. Confrontational outreach actions go in *3.2 Protests & Civil Disobedience.* |
|  |  | *briefing newspaper reporters on conservation issues, Facebook campaigns, public service radio ads, zoo exhibits about threats to animal habitat, recording conservation songs, producing puppet shows with conservation message, door-to-door engagement, taking children on nature walks* |  |  |  |
|  | **3.2 Protests & Civil Disobedience** | | Organizing or engaging in... 1 Protests (confrontations or refusing to engage) 2 Public identification of wrong-doers 3 Impeding activities (legal / illegal passive civil disobedience) 4 Sabotage (illegal active monkeywrenching) | Promoting desired awareness and subsequent desired behavior change by conducting protests, naming and shaming, civil disobedience, or sabotage activities | Activities that seek to draw attention to and/or impede various conservation threats or drivers of threats. In general, these activities seek to put pressure on the actors responsible for the threats and/or make it too expensive to continue with the threat behaviors. Note that inclusion of illegal actions in this classification explicitly does NOT constitute an endorsement of these tactics. |
|  |  | *organizing protest marches against oil drilling, investigative journalism naming & shaming polluting companies, impeding whaling vessels, sitting in trees to prevent logging, sabotaging wildlife traps* |  |  |  |
| **4. Law Enforcement & Prosecution** | | |  | Actions monitoring and enforcing compliance with existing laws and policies at all levels to deter threats or compel conservation action | Whereas *7. Legal & Policy Frameworks* is about creating laws and policies to support conservation, this class contains actions implementing and enforcing these laws and policies at all levels ranging from global treaties to local community or tribal customary rules. |
|  | **4.1 Detection & Arrest** | | Reducing or deterring illegal behaviors through... 1 Surveillance 2 Patrolling 3 Guarding checkpoints / borders 4 Carrying out investigations 5 Establishing/maintaining informer networks 6 Arrest & interdiction | Detecting, directly stopping, and/or deterring violations of existing laws and policies | Interdiction activities are designed to both stop existing law breakers as well as deter future law breakers from illegal and generally criminal activities. Interdiction activities can take place at any point along the transactional chain that links resource harvesters or poachers, traders, financers or other middlemen, and end consumers. |
|  |  | *monitoring wildlife trafficking across borders, investigating reports of illegal grazing, setting up informer network against tiger poaching, interdicting an illegal fishing vessel* |  |  |  |
|  | **4.2 Criminal Prosecution & Conviction** | | Deterring threat behaviors through... 1 Prosecuting alleged crimes 2 Trying alleged crimes 3 Punishing proven crimes (prisons, fine collection, rehabilitation) | Ensuring appropriate application of sanctions for violations of existing laws and policies | Activities designed to ensure that appropriate sanctions are meted out and that laws will thus have the desired deterrence effect. However, actions that are primarily designed to teach prosecutors how to do their jobs could go in *9.2 Training & Capacity Development* and actions that are primarily designed to create or improve courts or prisons could go in *10.2 Institutional & Civil Society Development* since these are more enabling condition actions. |
|  |  | *collecting evidence to prosecute a wildlife trader, holding trials for alleged law breakers, monitoring to ensure criminals serve time and pay fines* |  |  |  |
|  | **4.3 Non-Criminal Legal Action** | |  | Threatening or bringing non-criminal legal action to get individuals, organizations, agencies or firms to change or deter undesired behaviors or compel conservation action | Non-criminal legal activities designed to change or deter undesired behaviors or compel desired conservation action. Activities can be initiated by relevant government agencies using their statutory authority, or by various actors through civil legal proceedings. Includes processes in which government environmental agencies are authorized to officially review or comment on policies or projects (eg highway construction or new housing developments) implemented by other agencies or private sector actors. |
|  |  | *suing an agency to take action to protect an endangered species, suing a company to stop illegal logging, citing homeowners for sewage violations, agency review of policies or projects* | Changing behavior through... 1 Civil law suits 2 Agency enforcement 3 Agency or judicial review |  |  |
| **5. Livelihood, Economic & Moral Incentives** | | |  | Actions using livelihood, other economic and moral incentives to directly influence attitudes and behaviors | This class contains actions that use positive or negative incentives to promote desired behavior change. |
|  | **5.1 Linked Enterprises & Alternative Livelihoods** | | Creating incentives to change behaviors through... 1 Linked product-producing enterprises (consumptive) 2 Linked ecological service-using enterprises (non-consumptive) 3 Non-linked enterprises & livelihoods | Developing enterprises that directly depend on the maintenance of natural resources or provide substitute livelihoods as a means of changing attitudes and behaviors | Both linked enterprises and alternative livelihoods involve providing income and/or subsistence resources to natural resource users. Linked enterprises depend on the natural resource base and thus provide an incentive to local stakeholders to use these resource sustainably over the long-term. Alternative livelihoods attempt to find a substitute for a damaging resource-based livelihood (eg unsustainable fishing or logging). |
|  |  | *non-timber forest product harvesting business, wild salmon fishery, subsistence hunting & gathering, training loggers to be ecotourism guides, training loggers for factory jobs* |  |  |  |
|  | **5.2 Better Products & Management Practices** | | Changing behaviors by... 1 Developing better products & practices 2 Promoting better products & practices 3 Providing better products 4 Providing training or technical assistance for better practices 5 Removing barriers to adoption of better products or practices | Developing, promoting and/or providing more environmentally-friendly products or practices that substitute for environmentally damaging ones | In Version 1.0, this category primarily focused on finding substitutes for environmentally damaging products and behaviors. This category has now been expanded to encompass environmentally better products and management practices. Although many people refer to "best" management practices, we use the term "better" to show that practices can always be further improved. There is some overlap with *5.3 Market-Based Incentives* since certification systems are often designed to incent or promote better management practices among service providers or commodity producers. This category, however, is more about developing, promoting, providing, and/or removing barriers (eg risk minimization) to adoption of better products and practices. |
|  |  | *competition to develop sea turtle friendly fishhooks, research to develop low water-use crops, providing access to weed-free hay, setting up recycling services, training in chicken production as a substitute protein source for bushmeat, setting up grass & forest banking systems to mitigate farmer risk, engagement with corporations to "green" their supply chains* |  |  |  |
|  | **5.3 Market-Based Incentives** | | Creating incentives to change behaviors through... 1 "Green" certification of products or services 2 Boycotts of "non-green" products or services 3 Environmental markets (eg CO2 emissions, water) 4 "Green" financing (eg bank loans, divestiture) | Using market mechanisms to change behaviors and attitudes | Activities using market forces to change attitudes and behaviors. It is closely aligned with *5.4 Direct Financial or Economic Incentives,* with the difference being that in this case, the implementer does not have to raise the funds to make the conservation payments, but is instead relying on market forces to drive the system. |
|  |  | *promoting responsible palm oil production, educating consumers about seafood sustainability, boycotts of non-dolphin safe tuna, creating carbon market for forest conservation, providing loan guarantees to green businesses, campaign to get universities to divest from fossil fuel companies* |  |  |  |
|  | **5.4 Direct Economic Incentives** | | Creating incentive to change behaviors through... 1 Direct payments or subsidies for desired behaviors 2 Taxes on undesired behaviors 3 Valuation of ecological services 4 Monetary awards & prizes | Using direct or indirect payments or ascribing economic value to change behaviors and attitudes | Activities using non-market based financial and economic incentives to change behavior. In this case, the implementer has to raise ongoing funds needed to make conservation payments or provide subsidies and also needs to ensure that the subsidies go to the right people. It is also possible to have financial dis-incentives in the form of taxes. There may be a research component to Valuation of Ecological Services. Awards & prizes could also be seen as an outreach strategy, since they often seek to draw attention to conservation work and issues as much or more than they directly incent conservation behaviors. |
|  |  | *compensation payments for elephant crop-damage, giving or raising money for "green" political candidates, providing medical clinic in return for conservation, tax incentives to promote conservation, punitive taxes on high fuel consuming cars, using economic valuation of wetland flood protection services to promote wetland conservation, prizes for environmental work* |  |  |  |
|  | **5.5 Non-Monetary Values** | | Creating incentives to change behaviors by appealing to... 1 Health & social service benefits 2 Security benefits 3 Spiritual / moral / cultural benefits | Using intangible and moral values to change behaviors and attitudes | There is some overlap between this category and *3.1 Outreach & Communications* since it seems likely that actively promoting these intangible incentives would require some sort of outreach strategy. Research to determine values could go here or in *8.1 Basic Research & Status Monitoring.* |
|  |  | *linking conservation to human health, linking conservation to food security, developing religious or cultural arguments for conservation* |  |  |  |
| **C. ENABLING CONDITION ACTIONS** | | |  | Actions to create the conditions necessary for other conservation efforts to succeed | These actions generally require other actions to be completed in order to ultimately conserve the target(s). |
| **6. Conservation Designation & Planning** | | |  | Actions directly protecting sites and/or species | This class contains all actions related to establishing direct protection of sites and species. It technically could be a subset of *7. Legal & Policy Frameworks,* but is so central to conservation that it gets its own entry. Direct management actions for sites and species go in Classes 1 and 2. |
|  | **6.1 Protected Area Designation &/or Acquisition** | | Establishing or demarcating... 1 Government protected area 2 Private protected area 3 Community or tribal natural resource use area | Legally or formally establishing or expanding public or private parks, reserves, and other protected areas roughly equivalent to IUCN Categories I-IV | This category covers the establishment of protected areas; ongoing management of the protected areas involves using other actions listed in this classification. |
|  |  | *gazetting a national park, demarcating a town wildlife sanctuary, purchasing a land trust property, establishing tribally owned hunting grounds* |  |  |  |
|  | **6.2 Easements & Resource Rights** | | Purchasing or promoting... 1 Conservation easements 2 Specific resource rights | Legally or formally establishing protection of some specific aspect of the natural resources on public or private lands | This category focuses on establishing protection over one or more resources on site, rather than the overall site itself. Easements that provide tax breaks other financial incentives to landowners could be classified as *5.4 Direct Economic Incentives*, but are here to show their link to land and resource protection. |
|  |  | *convincing landowners to establish easement restricting development rights, purchasing water/instream flow rights, securing resource tenure rights for local communities* |  |  |  |
|  | **6.3 Land/Water Use Zoning & Designation** | | Conducting or doing... 1 Land-use zoning (where to put plantations, housing) 2 Conservation area designation (beyond protected areas) | Designating land/water uses or designating conservation areas outside of IUCN Categories I-IV | This category includes both land/use planning and zoning (which may or may not have legal standing) as well as designating conservation areas that have limited or even no legal protection, and thus are aimed at attracting conservation attention to the area. |
|  |  | *zoning development vs conservation areas, designating a wild & scenic river or an important bat conservation area (without formal protection)* |  |  |  |
|  | **6.4 Conservation Planning** | | Planning for managing... 1 Ecoregions or large land/seascapes 2 Sites/protected areas 3 Species/taxonomic groups 4 Thematic projects and programs | Planning for management of sites, species, or thematic conservation projects | This category involves actions needed to iteratively design and plan for the management of sites, species and other taxa, and thematic projects and programs. Thematic projects are non-site-based or species-based (eg stopping illegal wildlife trade). This category includes planning and oversight of conservation activities, but not implementation of these activities (which are all the other actions in this classification). There is a fuzzy line between planning (this category) and the iterative adaptive management that results from the work in *8.2 Evaluation, Effectiveness Monitoring & Learning.* |
|  |  | *developing a management plan for a national park, revision of an existing plan for a migratory bird species across its range, spatial planning and prioritization effort to identify important bat areas, planning a livelihood project* |  |  |  |
|  | **6.5 Site Infrastructure** | | Building or maintaining infrastructure to support site... 1 Protection (eg border fences, patrol huts) 2 Direct management (eg greenhouses, supply sheds) 3 Resource extraction (eg sawmill, fish processing building)  4 Transport (eg roads, airstrips)  5 Tourism & recreation (eg visitor centers, boat ramps) 6 Learning & research (eg student dorms, labs)  7 Administration (eg headquarters, power plants) | Building or maintaining the physical infrastructure for protected areas and other conservation sites | This category is primarily about building and maintaining capital investments; expenditures on routine maintenance belong in 1.1. |
|  |  | *creating guard posts, border fences, roads, recreational areas* |  |  |  |
| **7. Legal & Policy Frameworks** | | |  | Actions developing and influencing legislation, policies and voluntary standards affecting conservation | This class contains all actions related to the development of conservation laws and polices except those in *6. Conservation Designation.* Note that different societies and legal systems use words like "regulations" and "policies" in different ways. |
|  | **7.1 Laws, Regulations & Codes** | | Creating, amending or influencing environment-related... 1 International law, conventions & treaties 2 National law or regulations 3 State/provincial law or regulations 4 Municipal law or regulations 5 Tribal law & formal customs 6 Private sector/civil society codes 7 Cross-sectoral | Creating, amending, or influencing laws, regulations and codes at all levels | Laws and regulations refers to the official legal code governing society – what some people refer to as "hard law." Private sector/civil society codes are formal agreements among various organizations. Different actors play different roles in the overall legislative process. For example, only government agencies enact public legislation, but NGOs and other actors can write draft legislation, educate or lobby lawmakers, and otherwise try to influence the legislative process. |
|  |  | *advocating for strengthening international wildlife trade laws, writing national legislation for endangered species conservation, proposing state or provincial ballot initiatives to fund conservation, making the case for town sewage regulations, enacting tribal laws governing hunting seasons, creating a roundtable of key soy producers to create standards for responsible soy production* |  |  |  |
|  | **7.2 Policies & Guidelines** | | Creating, amending or influencing environment-related... 1 International policies 2 National polices 3 State/provincial policies 4 Municipal policies 5 Tribal policies 6 Private sector/civil society policies 7 Cross-sectoral | Creating, amending, or influencing policies and guidelines at all levels | Policies and guidelines govern how legislation, regulations or codes are implemented – what some people refer to as "soft law." |
|  |  | *advocating for agency implementation of international wildlife trade laws, writing national policies for endangered species conservation, serving on agency-NGO committee to determine hunting permitting processes, providing support to tribal leaders on environmental matters* |  |  |  |
| **8. Research & Monitoring** | | |  | Actions collecting data and transforming it into information to support conservation work | This class contains conservation actions that primarily involve research and monitoring that build the knowledge base needed to support conservation. |
|  | **8.1 Basic Research & Status Monitoring** | | Conducting research or analysis in different disciplines on... 1 Biological targets 2 Human wellbeing targets 3 Threats / biophysical factors 4 Socioeconomic drivers | Collecting, managing and analyzing data and creating information about any conservation-related factors | Research that contributes to basic understanding of the situations in which conservation takes place, independent of any specific conservation actions. It also includes to data collection to assess the status of key factors in these situations. This category includes all aspects of the research process including writing or reviewing proposals, developing protocols and methods, collecting data, analyzing data, creating or maintaining data storage and aggregation tools, peer reviewing results, and sharing and disseminating findings. |
|  |  | *biological research on the distribution of an endangered bird, setting up a database/scorecard on the status of wetlands in a region, modelling climate change effects, surveying income and attitudes of local community members* |  |  |  |
|  | **8.2 Evaluation, Effectiveness Measures & Learning** | | Collecting information about conservation work... 1 Specific projects 2 Cross-project or program comparisons 3 Discipline-level frameworks (double loop learning) | Assessing and learning about the effectiveness of conservation work | Collecting data and creating information related to the effectiveness of specific conservation actions at all scales. This category includes all aspects of the research process including writing or reviewing proposals, developing protocols and methods, collecting data, analyzing data, creating or maintaining data storage and aggregation tools, peer reviewing results, and sharing and disseminating findings. |
|  |  | *formative evaluation of a conservation project, conducting a systematic review of the effectiveness of an action, creating a database of conservation projects, developing standard classifications* |  |  |  |
| **9. Education & Training** | | |  | Actions enhancing the knowledge and skills of specific individuals | This class contains actions that build individual capacity to do good conservation. Traditional "environmental education" is an awareness / behavior change strategy and is thus in *3.1 Outreach & Communications.* |
|  | **9.1 Formal Education** | | Providing conservation courses, modules or materials for... 1 Primary education 2 Secondary education 3 College or university education 4 Adult / continuing education | Enhancing knowledge and skills of students in a formal degree program | Formal education seeks to build the long-term conservation capacity of students. A specific strategy of providing general education to resource users so that they have non-resource using livelihood alternatives should be included in *5.1 Linked Enterprises & Alternative Livelihoods*. |
|  |  | *creating a high school environmental course, teaching a conservation module in a university course, writing a conservation text book* |  |  |  |
|  | **9.2 Training & Individual Capacity Development** | | Providing conservation capacity development through... 1 Hands-on coaching & technical assistance 2 Workshops & professional development training courses 3 Developing training materials (manuals, software, videos) | Enhancing knowledge, skills and information exchange for practitioners, stakeholders, and other relevant individuals in structured settings outside of degree programs | Providing practitioners with specific knowledge and skills. Coaching involves more hands-on work with practitioners as they actually implement conservation activities whereas training is more about providing basic knowledge and skills that will be applied at a later date. There is a fine line between providing technical assistance as primary strategy vs providing technical assistance as one activity within another action such as *5.2 Better Products & Management Practices.* |
|  |  | *coaching a team developing a strategic plan, providing technical assistance to landowners to use better practices, training course in proscribed fire, writing how-to manuals for project managers, elders sharing traditional ecological knowledge* |  |  |  |
| **10. Institutional Development** | | |  | Actions creating the institutions needed to support conservation work | This class contains actions that build institutional capacity to do good conservation. The term "organization" is used to encompass all types of institutions including government agencies, not-for-profits, firms and communities of practice. |
|  | **10.1 Internal Organizational Management & Administration** | | Establishing & managing conservation organizations... 1 Governance 2 Executive management 3 Human resources 4 Financial & legal management 5 Fundraising 6 Communications 7 Program / project management 8 Provision of org facilities & technology 9 Support functions (secretaries, guards, drivers) | The basic work needed to establish and operate conservation organizations | This category covers all the basic functions needed to support one's own organization. |
|  |  | *hiring and managing staff for protected areas or conservation agencies, serving on the board of a conservation organization, managing a conservation program, providing basic support functions for a reserve* |  |  |  |
|  | **10.2 External Organizational Development & Support** | | Establishing & supporting organizations through... 1 Direct organizational support (consulting / volunteering) 2 Organizational establishment & incubation 3 Providing association / membership services (LTA, AZA) 4 Developing / providing organizational management tools | Creating or providing non-financial support & capacity building for conservation organizations | This category covers most non-financial work involved in supporting other organizations to do conservation work. There is a fine line between providing technical assistance *(9.2 Training & Capacity Development)* which focuses primarily on individuals and project teams versus this category which focuses primarily on entire programs and organizations. |
|  |  | *providing consulting services to a conservation organization, international volunteers or circuit riders helping to develop organizational capacity, work to create and strengthen courts that can prosecute wildlife crimes, helping catalyze and incubate formation of a new land trust, developing work planning software for conservation organizations* |  |  |  |
|  | **10.3 Alliance & Partnership Development** | | Creating or maintaining partnerships focused on... 1 Coordinating conservation implementation 2 Knowledge generation & sharing | Forming and facilitating partnerships, alliances, and networks of organizations | This category encompasses work to create cross-organizational conservation institutions. Many of these partnerships provide support to their members so there is a fine line between *10.2 External Organizational Development & Support* and this category; the former has supporting individual organizations as its primary focus whereas the latter is more about maintaining the network. |
|  |  | *convening meetings of local stakeholders in a community reserve, an international forum to share information about wildlife crimes, a membership services association of land trusts, a regional learning network, an international academic society* |  |  |  |
|  | **10.4 Financing Conservation** | | Providing funds for conservation including...  1 Member / small contributions 2 Unrestricted grants 3 Restricted grants (eg scholarships, for a specific project) 4 Program related investments / soft loans 5 Commercial loans 6 In-kind services 7 Financing mechanisms (eg debt-for-nature swaps) | Raising and providing funds for conservation work | This category includes work aimed primarily at providing funding for conservation work. Obviously, almost every other action has some component of financing. There is some potential overlap with *5.4 Direct Economic Incentives* and this category; with the difference being that the former is targeted to incenting specific behaviors, whereas the latter is about generally providing the funds required to take on other conservation actions. |
|  |  | *door-to-door fundraising efforts, private foundation or government grants, foundation investments in green businesses, corporate philanthropy, national debt-for-nature swaps* |  |  |  |
